# Identification of Pre-Erythrocytic Malaria Antigens That Target Hepatocytes for Killing *In Vivo* and Contribute to Protection Elicited by Whole-Parasite Vaccination

**DOI:** 10.1371/journal.pone.0102225

**Published:** 2014-07-15

**Authors:** Lin Chen, Gladys J. Keitany, Xiaohong Peng, Claire Gibson, Isaac Mohar, Marissa Vignali, Ian N. Crispe, Fusheng Huang, Ruobing Wang

**Affiliations:** 1 Department of Pathogenic Biology, Third Military Medical University, Chongqing, China; 2 Seattle Biomedical Research Institute, Seattle, Washington, United States of America; 3 Department of Pathology, University of Washington, Seattle, Washington, United States of America; 4 Department of Immunology, University of Washington, Seattle, Washington, United States of America; 5 Department of Global Health, University of Washington, Seattle, Washington, United States of America; Museum National d'Histoire Naturelle, France

## Abstract

Pre-erythrocytic malaria vaccines, including those based on whole-parasite approaches, have shown protective efficacy in animal and human studies. However few pre-erythocytic antigens other than the immunodominant circumsporozoite protein (CSP) have been studied in depth with the goal of developing potent subunit malaria vaccines that are suited for use in endemic areas. Here we describe a novel technique to identify pre-erythrocytic malaria antigens that contribute to protection elicited by whole-parasite vaccination in the mouse model. Our approach combines immunization with genetically attenuated parasites and challenge with DNA plasmids encoding for potential protective pre-erythrocytic malaria antigens as luciferase fusions by hydrodynamic tail vein injection. After optimizing the technique, we first showed that immunization with *Pyfabb/f^−^*, a *P. yoelii* genetically attenuated parasite, induces killing of CSP-presenting hepatocytes. Depletion of CD8^+^ but not CD4^+^ T cells diminished the killing of CSP-expressing hepatocytes, indicating that killing is CD8^+^ T cell-dependent. Finally we showed that the use of heterologous prime/boost immunization strategies that use genetically attenuated parasites and DNA vaccines enabled the characterization of a novel pre-erythrocytic antigen, Tmp21, as a contributor to *Pyfabb/f^−^* induced protection. This technique will be valuable for identification of potentially protective liver stage antigens and has the potential to contribute to the understanding of immunity elicited by whole parasite vaccination, as well as the development of effective subunit malaria vaccines.

## Introduction

Malaria remains a daunting public health challenge in spite of global elimination efforts that have significantly reduced incidence and death caused by this parasitic disease. An estimated 2.5 billion people are at risk of infection, which causes ∼250 million clinical cases and ∼840,000 child deaths per year in sub-Saharan Africa [1]. Since all clinical symptoms derive from the blood stages (BS), malaria vaccines that block parasite development during pre-erythrocytic (PE) stages prevent all human disease symptoms [2]. With up to 100% efficacy in human trials, live attenuated whole-parasite vaccines have been most effective to date, and include sporozoites that have been radiation-, drug-, or genetically-attenuated (reviewed in [3]). All of these can invade hepatocytes but subsequently arrest at different points during the liver stage (LS) or early in the BS of the life cycle of the parasite, while simultaneously inducing immune responses that protect against subsequent challenge with *wild type* sporozoites (*wt* spz). For example, by knocking out genes that are essential for LS parasite development, genetically attenuated parasite (GAP) vaccines have been shown to induce sterile and long-lasting protective immunity against challenge with *wt* spz in mice [4]–[6]. Similarly, immunization through the bite of mosquitos infected with *P. falciparum* or *P. vivax* irradiation-attenuated sporozoites (irr-spz) can protect humans from infection after challenge with *wt* spz [7]–[9]. Importantly, the PfSPZ vaccine was recently reported to protect 80% of volunteers who received 4–5 doses of intravenously administered irr-spz [10], in line with the vaccine efficacy required for eradication as per recent WHO guidelines [11].

In spite of their promise, currently available whole-parasite malaria vaccines require inoculation with as many as 1,000 bites of *P. falciparum*-infected mosquitoes, or intravenous administration of high doses of purified, cryopreserved irradiated sporozoites. Thus, a rational malaria vaccine development plan should also include subunit vaccines, which circumvent the logistical constraints of generating and administering live attenuated parasites and thus constitute a complementary approach that could be used to boost and maintain protective immunity elicited by live-attenuated parasite vaccinations. However, the most advanced subunit malaria vaccine, RTS,S, which is based on the immunodominant circumsporozoite protein (CSP), only exhibited a 32–50% anti-disease (but not anti-infection) efficacy among African children in Phase IIIb trials [12], [13].

Animal model studies and human clinical trials have demonstrated that CD8^+^ T cells play an important role in protection induced by live-attenuated sporozoite vaccination [10], [14]–[19]. In particular, we have used *in vitro* live-cell imaging to demonstrate that cytotoxic CD8^+^ T cells from mice immunized with *Py*GAP can directly kill LS parasite-infected hepatocytes [14]. In addition, antigen-specific CD8^+^ T cells have also been shown to correlate to protection induced by subunit vaccines [20]–[22].

Immune responses elicited by vaccination with whole parasites are biased towards CSP; however, several studies have shown that protection against malaria parasites can be achieved in the absence of CSP [23]–[25]. Despite these observations, few PE antigens other than CSP have been evaluated as vaccine candidates, including LSA1, CelTOS and TRAP [26]. The identification of novel PE antigens is hindered by the difficult culturing of infected primary hepatocytes. A recent attempt to expand the repertoire of PE antigens characterized antigen-specific IFN-γ production by splenic lymphocytes in CSP-tolerant mice immunized with irr spz [27]. The authors concluded that although immunization with several non-CSP antigens generated large numbers of specific CD8^+^ T cells that produced IFN-γ levels comparable to those elicited by CSP, only CSP was able to protect mice against challenge with wt spz. Another recent study aimed at identifying pre-erythrocytic antigens conducted a systematic profiling of H2b-restricted *P. berghei* peptides recognized by CD8^+^ T cells from mice immunized with whole malaria parasites [28]. *Pb*S20 and *Pb*TRAP were identified as targets of CD8^+^ T cells although only TRAP-specific cytotoxic CD8^+^ T cells were shown to contribute to protection against sporozoite challenge. These results support the importance of developing new methods to identify PE antigens that contribute to vaccine protection.

We report the optimization of a technical approach that characterizes the ability of antigen-specific CD8^+^ T cells to eliminate hepatocytes *in vivo* following immunization with whole parasite vaccines as a means to validate potential vaccine candidates. This method combines the use of Hydrodynamic Tail Vein Injection (HTVI) to deliver naked DNA encoding luciferase-tagged malaria LS antigens directly to the liver [29]–[31] with an *in vivo* imaging system (IVIS) that allows real-time monitoring of the abundance of the luciferase-tagged antigens in the liver [32]. After validating this method in the *P. yoelii* murine immunization/challenge model using CSP as a positive control, we used it to confirm that a potential new LS antigen, *Py*Tmp21, which reduces liver stage burden *in vivo*, contributes to the protection elicited by whole parasite vaccines. This technology will be useful to down-select candidate LS antigens.

## Materials and Methods

### Animal and ethics statement

Five to ten week-old female BALB/c mice were obtained from Jackson Laboratory. All murine studies and procedures were approved by the institutional Animal Care and Use Committee of the Seattle Biomedical Research Institute (Seattle BioMed) following the NIH guidelines for animal housing and care.

### Construction of DNA plasmids

phCMV-*Py*CSP-Luc was constructed by cloning an amino-terminal Myc-tagged synthetic *P. yoelii* CSP gene fragment (IDT) containing a CD4^+^ epitope (aa 57–70), 3 units of the central repeat (aa 139–156), and the carboxy-terminus (aa 280–345) of CSP into the phCMV-Luc vector as a carboxy-terminal fusion with the firefly luciferase reporter gene using restriction enzymes XhoI and HindIII (Figure S1A).

phCMV-*Py*Tmp21-Luc was constructed by cloning an amino-terminal Myc-tagged fragment of transmembrane protein *Py*Tmp21 (*Plasmodium yoelii yoelii* str. 17XNL PY06414, PlasmoDB, aa 26 to 181) excluding the amino-terminal endoplasmic reticulum targeting signal sequence and the carboxy-terminal transmembrane domain that was PCR amplified from *P. yoelii* cDNA using the following primers: F *Py*Tmp21: 5′-CGATCTCGAGATGGAACAAAAACTCATCTCAGAAGAGGATCTGATATATTTATCCT TAAAACC-3′ and R *Py*Tmp21 5′-CGTAAAGCTTGCTAATGTATCATTTAA TTTTTCG-3′ (Figure S1A). After confirming the orientation of the inserts by PCR and double digest with XhoI and HindIII restriction enzymes, positive clones were sequenced to ensure accurate amplification and in-frame cloning with the luciferase open reading frame. Plasmid DNA was prepared by using the Qiagen EndoFree Mega Plasmid Kit (Qiagen, Valencia, CA). Full-length *Py*Tmp21 was PCR amplified from non-lethal *P. yoelii* 17X NL clone 1.1 and the resulting PCR product was cloned into the gWIZ vector (Gentlantis, CA, USA) using SalI and NotI restriction sites (McLab, CA, USA).

### Luciferase activity assay

COS-7 cells (obtained from the American Type Culture Collection, ATCC) were cultured in DME medium supplemented with 10% fetal bovine serum, 2 mM L-glutamine and 100 U of penicillin-streptomycin/ml. One day before transfection, 1×10^6^ cells were plated in 10 cm^2^ plates in growth medium without antibiotics. Twenty-four hours later, a total of 4 µg of DNA was transfected with lipofectamine 2000, following the manufacturer's instructions (Invitrogen). After 48 hours, cells were collected and lysed for luciferase expression analysis. Protein concentration was determined using a Bradford protein assay kit (Bio-Rad). The luciferase activity of cell lysates was measured using the Bright-Glo luciferase assay system (Promega). Luminescence was measured on a CentroXS3 LB 960 luminometer. Measurements were taken in triplicate. Light emission was integrated over a 10-second time period.

### Western Blot analyses

Twenty µg of total protein from transfected COS-7 cell lysates containing protease inhibitor (Roche) were resolved by electrophoresis on 12% SDS-PAGE gels (Bio-Rad), and transferred to a nitrocellulose membrane using a wet transfer cell apparatus (Bio-Rad). The membrane was blocked for 2 h in TBS buffer containing 0.05% Tween 20 (TBS-T) and 5% not-fat dry milk and incubated at 4**°**C overnight in a 1∶1,000 dilution of anti-luciferase antibody (GeneTex) in 5% milk/TBS-T. Next, the membrane was washed in TBS-T, incubated for 2 h with 1∶20,000 dilution of goat anti-mouse IgG-HRP in 5% milk/TBS-T, and detected by enhanced chemiluminescence (ECL plus) according to the manufacturer's instructions.

### Whole-parasite immunization strategies

The genetically attenuated parasite *P. yoelii fabb/f^−^* (*Pyfabb/f^−^*), which carries a targeted deletion of FabB/F, a critical enzyme in fatty acid synthesis and therefore arrests late in the liver phase of the malaria life cycle [19], was isolated from the salivary glands of infected *A. stephensi* mosquitoes.

For the first group of experiments, BALB/c mice were vaccinated intravenously with two doses of either 50,000 *Pyfabb/f^−^* sporozoites or mosquito salivary gland debris two weeks apart. For the heterologous immunization strategies, mice were vaccinated 3 times by intramuscular injection with 20 µg of gWIZ/*Py*Tmp21 recombinant plasmid DNA, followed by boosting with a single dose of *Pyfabb/f^−^* sporozoites, or, alternatively with a single dose of 50,000 *Pyfabb/f^−^* sporozoites followed by boosting with 20 µg of gWIZ/*Py*Tmp21 recombinant plasmid DNA.

### DNA vaccination and protection studies

BALB/c mice were vaccinated by administration of 20 µg of gWIZ recombinant plasmid DNA encoding for *Py*Tmp21, *Py*CSP or empty vector into the tibialis muscle at weeks 0, 3 and 6, using the Ichor Tri-GridTM delivery system (Ichor Medicals, CA, USA). Mice were challenged with 20,000 wild type *P. yoelii* sporozoites by i.v. injection 10 days after the third immunization. Inhibition of liver stage parasite development was measured by qRT-PCR at 42 hours post challenge [33]. The ratio of *P. yoelii* 18S RNA expression was compared to that of the mouse housekeeping gene GAPDH.

### Challenge by Hydrodynamic Tail Vein Injection

Ten week old BALB/c naïve mice or mice immunized as described above were injected with phCMV-Luc, phCMV-*Py*CSP-Luc or phCMV-*Py*Tmp21-Luc recombinant plasmid DNA by HTVI as previously described [29]. Challenge was performed 14 or 30 days after the immunized mice received the last dose of *Pyfabb/f^−^* sporozoites. In brief, mice were placed in a restrainer tube and injected rapidly in the tail vein within 5 seconds with approximately 10% volume/body weight of phosphate-buffered saline (PBS, Gibco, UK) containing 25 µg of plasmid DNA, using a 27 1/2 gauge needle.

### *In vivo* imaging of bioluminescence

Four hours, eight hours, 1 day, 2 days, 4 days and 7 days after HTVI, mice were anesthetized with 2% isoflurane and administered 150 mg/kg of luciferase substrate (D-luciferin, Xenogen-Caliper) intraperitoneally. Five minutes later, the mice were positioned in the imaging chamber (IVIS Lumina II) for data collection. Living Image (Xenogen-Caliper) software was used to measure photons emitted by region of interest (ROI), which were analyzed using the ICOR image analysis software. Data was quantified as photon sec/cm^2^/sr. Values are shown as mean ± SD.

### *In vivo* T cell Depletion

Mock-immunized mice and mice immunized with *Pyfabb/f^−^* sporozoites as described above were injected intraperitoneally with 0.5 mg of anti-CD8 monoclonal antibody 2.43 (TIB210; American Type Culture Collection), anti-CD4 monoclonal antibody GK1.5, or an equivalent dose of rat IgG2b isotype control for two consecutive days before being challenged with plasmid DNA by HTVI. The dose and regimen was optimized to deplete >95% of CD8^+^ T cells or CD4^+^ T cells (data not shown). Depletion of specific cell types was confirmed by surface staining of PBMC with Pacific Blue-conjugated anti-CD3, PerCP/cy5.5-conjugated anti-CD8 and APC-conjugated anti-CD4 antibodies (Biolegend) by flow cytometric analysis one day before challenge.

### Hepatocyte and lymphocyte purification

Twenty hours or seven days after mice were challenged with *Py*CSP-Luc recombinant plasmid DNA by HTVI, hepatocytes and infiltrating lymphocytes were harvested and purified as previously described [15]. Briefly, mouse livers were perfused with 10 mL of perfusion media (1X HBSS, 5 mM HEPES, and 0.5 mM EDTA) via the portal vein and treated *in situ* with 0.5 mg/mL collagenase in 10 mL of collagenase buffer (1X HBSS (without Ca/Mg), 0.5 mM CaCl, 0.5 mM MgCl, 5 mM HEPES). Afterwards, livers were collected and homogenized, and hepatocytes were isolated by low speed centrifugation at 500 rpm. The pellet was washed 3 times with R10 media (complete RPMI containing 10% fetal bovine serum (FBS), penicillin-streptomycin and glutamine) and suspended in 10 ml R10. Lymphocytes were isolated from the supernatant by centrifugation on a gradient of 44% Percoll buffer underlaid with 67% Percoll buffer (GE Life Sciences). Percoll gradients were centrifuged at 2,000 rpm, and mononuclear cells at the gradient interface were extracted, washed 3 times with R10, and suspended in complete FACs buffer (1X PBS containing 2% FBS).

### Intracellular staining and flow cytometry

For cytokine staining, 1×10^6^ liver lymphocytes were incubated for 6 hours at 37**°**C in the presence of Brefeldin A (10 mg/ml, Sigma-Aldrich) followed by 1 hour incubation with PE-Cy7-conjugated anti-CD3 antibody (Biolegend), PerCP/cy5.5-conjugated anti-CD8 antibody (Biolegend) and APC-labeled anti-H2Kd/CSP_280–288_ (SYVPSAEQI) tetramer antibody (obtained from the NIH tetramer core facility). Cells were subsequently washed with FACS buffer (1% BSA, 0.05% sodium azide in PBS), fixed and permeabilized by incubation at 4°C for 20 min in 100 µL BD Cytofix/Cytoperm (BD Biosciences)). After washing in Perm/Wash buffer (BD Biosciences), intracellular staining for IFN-γ was determined using a Pacific Blue-conjugated anti-IFN-γ clone XMG1.2 antibody (eBioscience).

To determine the subset and proportion of liver lymphocytes expressing *Py*CSP, 1×10^6^ lymphocytes were surface stained with BV421-conjugated anti-CD3 antibody, PerCP/Cy5.5-conjugated anti-B220 antibody, APC-conjugated Tie-2 antibody, APC-Cy7-conjugated anti-F4/80 antibody, PE-Cy7-conjugated CD11b antibody and BV605-conjugated CD11c antibody (BioLegend). Cells were washed and fixed as described above before being incubated with a polyclonal anti-rabbit PyCSP antibody (AbBiotech, Inc.) at a 1∶100 dilution in Perm/Wash buffer for 30 minutes at room temperature. After 3 washes with Perm/Wash buffer cells were incubated with Alexa-Fluor 488-conjugated anti-rabbit antibody for another 30 minutes. Cells were washed in Perm/Wash buffer and suspended in 120 µL of FACs buffer.

To determine the proportion of liver hepatocytes expressing *Py*CSP, 500 µL of cell suspension prepared as described above was pelleted and resuspended in 500 µL of Fixing Solution (1% Paraformaldehyde in PBS) for 10 minutes at RT. Cells were washed 3 times with FACs buffer before being stained with polyclonal rabbit anti-*Py*CSP antibodies as described above.

Data were acquired using a BD LSRII instrument and analyzed with FlowJo software (Tree Star, Inc.)

### Statistical analysis

All data are presented as mean ± SD. Differences between groups were determined by unpaired, two tailed Mann-Whitney tests. All p-values <0.05 were considered statistically significant.

## Results

### Plasmid construction and *in vitro* expression of a *P. yoelii* CSP luciferase fusion protein

We first established the HTVI/IVIS system in the *Pyfabb/f^−^* murine model, using CSP as a positive control. We constructed an amino-terminal Myc-tagged, carboxy-terminal luciferase fusion protein (*Py*CSP-Luc) by synthesizing a DNA fragment that encodes for a previously identified CD4 CSP epitope, followed by 3 copies of the central antigenic repeat and the carboxy-terminus of CSP, which has been shown to contain overlapping CD8 and CD4 epitopes [34]. This DNA fragment was cloned into the phCMV-Luc vector to generate phCMV*-Py*CSP-Luc (Figure S1A, left panel). To confirm the expression of the resulting fusion protein, we transfected COS-7 cells with either phCMV-Luc or phCMV*-Py*CSP-Luc, harvested these cells 48 h post transfection and prepared protein extracts. Both Western blot analysis using a polyclonal firefly luciferase antibody and a luminometry assay showed that *Py*CSP-Luc was expressed at a similar level to that of luciferase alone (Figure S1B and C).

### *In vivo* expression of *Py*CSP-Luc

**Figure 1:**
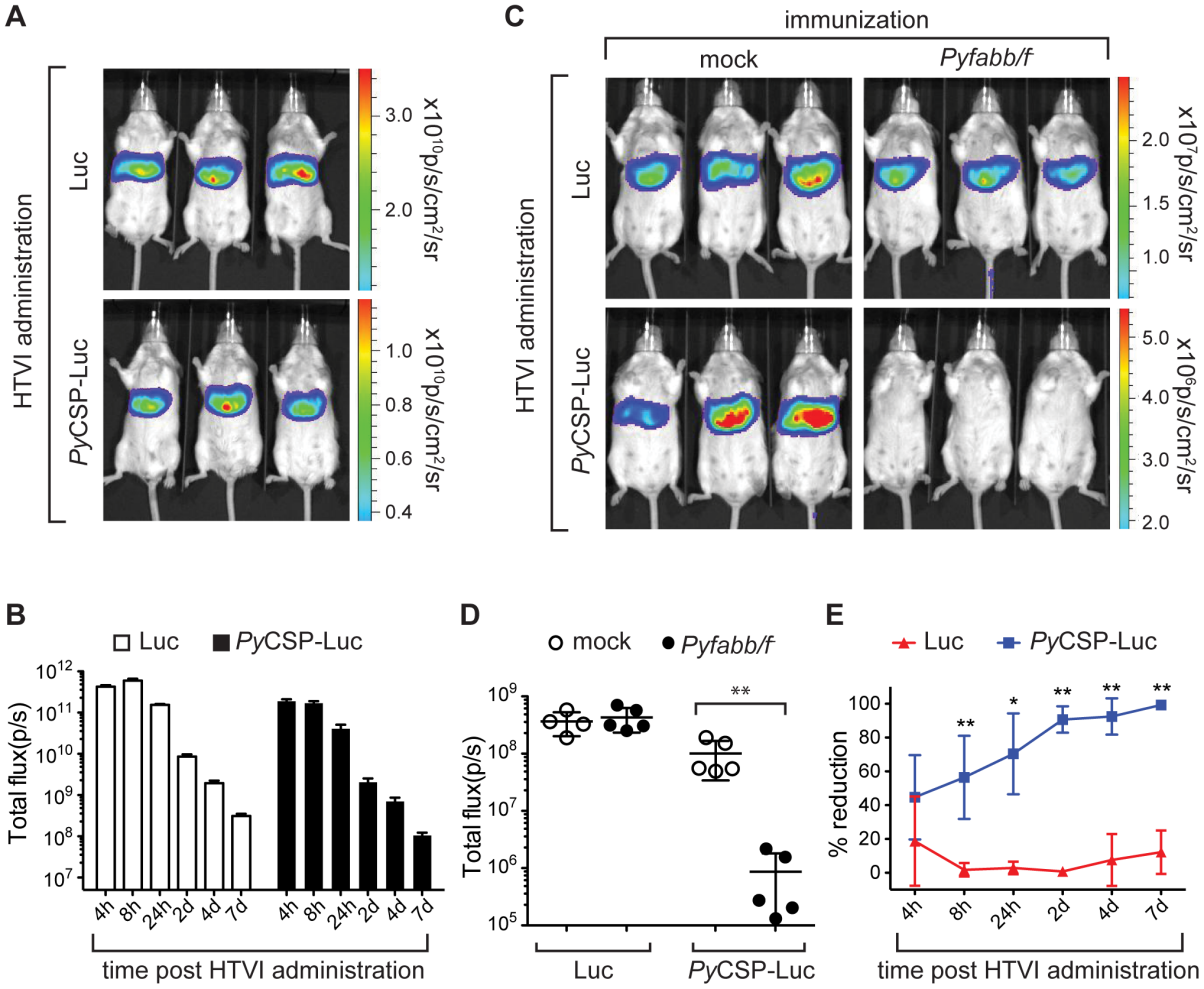
Immunization of mice with *Pyfabb/f^−^* reduces *Py*CSP-Luc *in vivo* luminescence. (A–B) Expression of luciferase (Luc) and *Py*CSP-Luc in the liver of mice immunized by HTVI. (A) Luciferase signal in live naïve BALB/c mice injected by HTVI with 25 µg of either phCMV-Luc plasmid DNA (top panel) or phCMV-*Py*CSP-Luc (bottom panel) and imaged 8 h later by IVIS, after injection of D-luciferin. The scale indicates radiance expressed as p/s/cm^2^/sr. (B) Kinetics of luciferase signal in live naïve BALB/c mice (shown as total flux p per second) during the course of the experiment. Data is representative of two individual experiments, with 3 mice each per group. (C–E) Reduction of luciferase signal in the liver of *Pyfabb/f^−^*-immunized mice upon HTVI challenge with *Py*CSP-Luc plasmid DNA (C) Luciferase signal in 3 representative live mice immunized twice with 50,000 *Pyfabb/f^−^* salivary gland sporozoites (right panel) or mock-immunized with salivary gland debris (left panel), challenged by HTVI with 25 µg of phCMV-Luc plasmid DNA (top panel) *Py*CSP-Luc (bottom panel) 30 days after the last immunization, imaged 7 d post challenge as described. Each group contained 4 to 5 mice. (D) Quantification of luciferase signal (shown as total flux p per second) from mice in the four groups described in part C. The data represents 4 to 5 individually analyzed mice in each group and correspond to mean ± SD; significant differences between the mean of the mock vs. *Pyfabb/f^−^*-immunized mice for each plasmid calculated using the Mann-Whitney test are indicated (** = *p*<0.01). (E) Inhibition of luciferase signal over the course of the experiment for mice challenged with plasmid DNA encoding for luciferase alone (Luc, red triangles) or *Py*CSP-Luc (blue squares), calculated as percentage reduction vs. the mock-immunization control). The data represents 4 to 5 individually analyzed mice in each group and correspond to mean ± SD; significant differences between the mean of Luc vs. *Py*CSP-Lus calculated using the Mann-Whitney test are indicated for each plasmid (* = *p*<0.005; ** = *p*<0.001).

To evaluate the expression of *Py*CSP-Luc fusion protein *in vivo*, we injected groups of BALB/c mice (n = 3) by HTVI with 25 µg of either plasmid DNA encoding for luciferase (Luc) or *Py*CSP-Luc fusion protein each, followed by administration of D-luciferase and whole-body bioluminescence detection by IVIS 4 h, 8 h, 24 h, 2 d, 4 d, and 7 d later. Using this approach, we detected high levels of luciferase activity in the liver of all mice in both groups as early as 4 h post HTVI, with expression peaking 4 to 8 h post HTVI and remaining high 7 d post HTVI (Figure 1A and B). This result shows that the *Py*CSP-luciferase fusion protein is expressed in a stable and persistent manner in the liver of mice injected with plasmid DNA through HTVI, and that by measuring whole-body bioluminescence *in vivo*, IVIS allows the real-time monitoring of the abundance of luciferase-fused tagged proteins.

Previous studies using microscopy of liver sections have reported that between 1 and 40% of hepatocytes express proteins encoded by plasmids administered by HTVI, and that the level of expression from non-parenchymal cells is negligible [29], [35]. We confirmed that hepatocytes correspond to the major liver cell type expressing *Py*CSP-Luc by isolating liver hepatocytes and lymphocytes 20 hours after administration of empty Luc vector or *Py*CSP-Luc by HTVI (Figure S2). Expression of luciferase in the liver was confirmed by IVIS before livers were perfused and extracted (data not shown). Using flow cytometry, we observed that between 7.5% and 31.5% of the hepatocytes in the animals that received *Py*CSP-Luc by HTVI expressed *Py*CSP, as compared to 1.6% of hepatocytes in animals injected with empty Luc vector (Figure S2A). In comparison, expression levels of *Py*CSP-Luc were similar between both groups of mice for the majority of the lymphocyte cell types tested, including Kupffer cells (F4/80^+^), dendritic cells (CD11b^+^/CD11c^+^), sinusoidal liver endothelial cells (Tie2^+^) and B cells (B220^+^). We only observed *Py*CSP expression in CD3^+^ T cells in one out of three mice that received *Py*CSP-Luc by HTVI. In conclusion, our data shows that the *Py*CSP-Luc plasmid is stably expressed in the liver for at least 7 days after HTVI administration, and is preferentially expressed by hepatocytes.

### Immunization of mice with *Pyfabb/f^−^* induces *Py*CSP-specific hepatocyte killing

Next, we determined whether the HTVI/IVIS method could be used as a tool to measure antigen-specific killing of hepatocytes upon whole parasite vaccination. To do this, mice were immunized intravenously (i.v.) twice, 14 days apart, with 50,000 *Pyfabb/f^−^* sporozoites dissected from the salivary glands of infected *A. stephensi* mosquitoes. As a control, mice were mock-immunized with salivary gland debris. Thirty days after the second dose, mice were challenged by HTVI with 25 µg of luciferase (Luc) or *Py*CSP-luc plasmid DNA, and bioluminescence was measured 4 h, 8 h, 24 h, 2 d, 4 d and 7 d later using IVIS. There was no significant difference in the kinetics of luciferase expression detected in the liver of *Pyfabb/f^−^* or mock-immunized mice challenged with plasmid encoding for luciferase (Figure 1C top panel and Figure 1D). However, the luciferase signal was dramatically decreased in *Pyfabb/f^−^*-immunized mice challenged with *Py*CSP-Luc plasmid DNA, as compared to mock-immunized mice (Figure 1C bottom panel and Figure 1D). Specifically, the luciferase signal was reduced by 45% 4 h post challenge, and continued to diminish over the course of the experiment, reaching a maximum reduction of 97% on d7 post challenge (Figure 1E). This result was highly reproducible, as very similar values were obtained in 3 independent experiments (data not shown).

The reduction in luciferase signal observed in the liver of mice upon HTVI challenge with *Py*CSP-Luc plasmid DNA after immunization with *Pyfabb/f^−^* suggests that vaccination with attenuated whole parasites induces specific killing of hepatocytes that express CSP epitopes [14], [17], [23], [36], [37]. Moreover, our data show that the combination of HTVI challenge with the non-invasive IVIS imaging technique constitutes a powerful tool that can be used to measure reductions of luciferase expression that are indicative of the elimination of specific antigen-presenting hepatocytes *in vivo*.

### CD8^+^ T cells are required for the elimination of *Py*CSP-Luc expressing liver cells in *Pyfabb/f^−^* immunized mice

**Figure 2:**
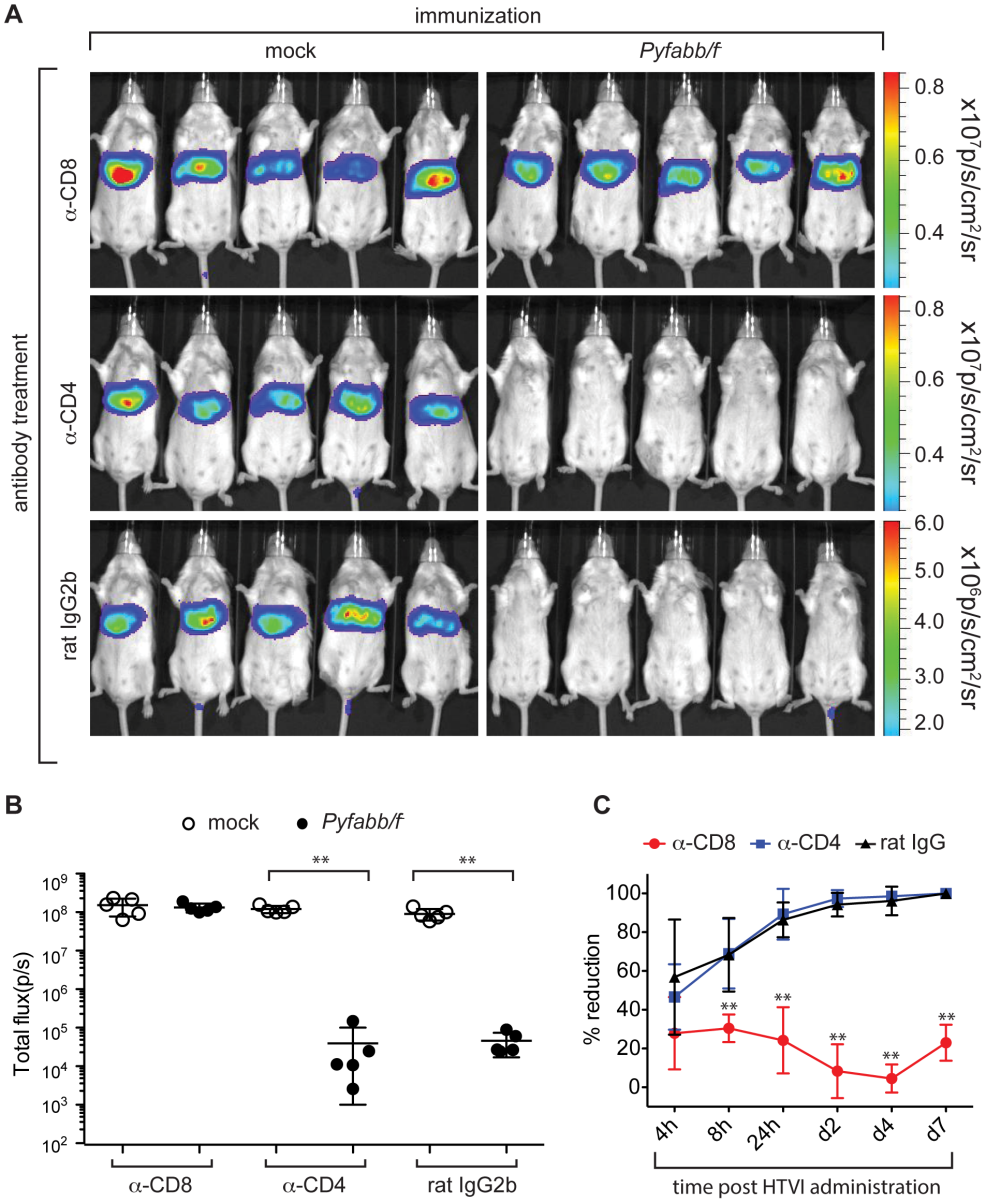
Elimination of hepatocytes that express *Py*CSP-Luc is mediated by CD8^+^ cells. (A) Luciferase signal in live mice immunized twice with 50,000 *Pyfabb/f^−^* salivary gland sporozoites (right panel) or mock immunized (left panel) with salivary gland debris, and treated 14 days later for two consecutive days with antibodies anti-CD8 (top panel), anti-CD4 (middle panels) or an equivalent amount of control rat IgG2b, before HTVI challenge with 25 µg of phCMV-*Py*CSP-Luc and imaged 7 d post challenge as described. Each group contained 5 mice. The scale indicates radiance expressed as p/s/cm^2^/sr. (B) Quantitation of the data shown in part A. Radiance is shown as total flux (p/s). The data represents 5 individually analyzed mice in each group and correspond to mean ± SD; significant differences between the mean of the mock vs. *Pyfabb/f^−^*-immunized mice for each treatment calculated using the Mann-Whitney test are indicated (** = *p*<0.01). (C) Kinetics of inhibition of luciferase expression upon depletion of CD8^+^ (red circles) or CD4^+^ (blue squares) T cells, or mock depletion (black triangles), calculated as percentage reduction compared to the mock immunization control for each condition. The data represents 5 individually analyzed mice in each group, and correspond to mean ± SD; significant differences between the CD8 or CD4 T cell depletion vs. the mock depletion are indicated (** = *p*<0.01).

We and others have previously shown that CD8^+^ T cells are critical for protection from challenge with *wt* spz after immunization of mice with whole parasite vaccinations [14], [15], [17], [37]. Thus, we investigated the role of CD8^+^ T cells in eliminating hepatocytes that present malaria specific antigens after immunization with *Pyfabb/f^−^*. To do this, we depleted CD4^+^ or CD8^+^ T cells in BALB/c mice immunized with *Pyfabb/f^−^* sporozoites (and in mock-immunized mice) thirteen days after the last dose of *Pyfabb/f^−^* through administration of monoclonal antibodies against CD4^+^ or CD8^+^ T cell receptors, or rat IgG2b as a negative control. Successful depletion of both CD4^+^ and CD8^+^ T cells was confirmed by analyzing the peripheral blood of individual mice by flow cytometry the day before challenge (data not shown). Twenty-four hours later, mice were challenged by HTVI with 25 µg of plasmid DNA encoding for *Py*CSP-Luc fusion protein. Depletion of CD8^+^ T cells (but not depletion of CD4^+^ cells nor treatment with rat IgG2b antibodies) abrogated the inhibition of luciferase signal induced by *Pyfabb/f^−^* immunization (Figure 2A–C). This result suggests that GAP immunization induces CSP-specific CD8^+^ T cells that are capable of eliminating hepatocytes that present CSP epitopes on their surface. Importantly, it agrees with *in vitro* data by our group and others showing that elimination of malaria-infected hepatocytes is mainly mediated by CD8^+^ T cells [14], [38].

### *Py*CSP-specific CD8^+^ T cells produce high levels of IFN-γ

**Figure 3:**
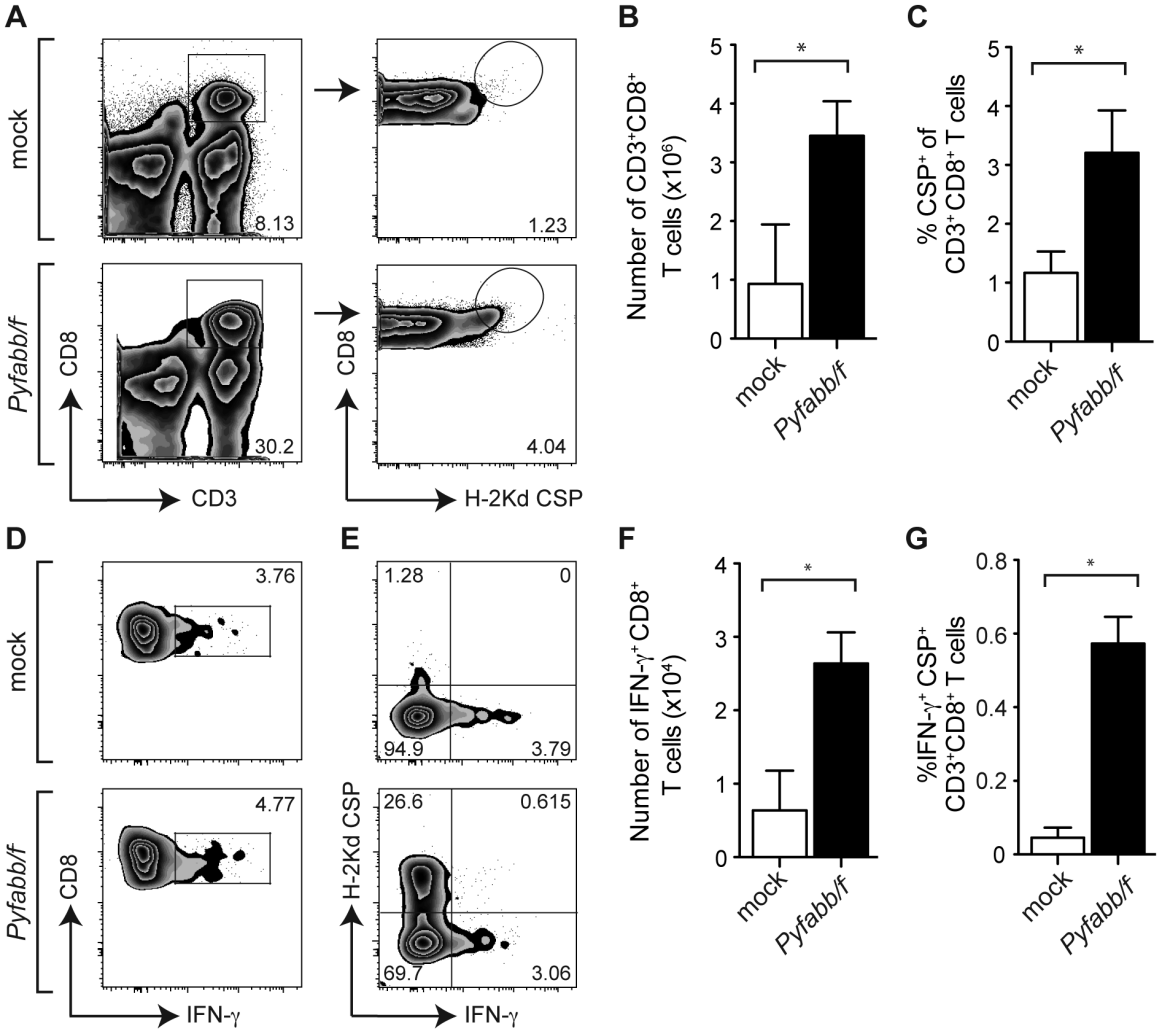
*Py*CSP-specific CD8^+^ T cells produce high levels of IFN-γ. (A) Characterization of infiltrating liver lymphocytes isolated from mice immunized with *Pyfabb/f* ^−^ sporozoites (bottom panel) or mock-immunized (top panel) 7 days after HTVI challenge with plasmid DNA encoding for *Py*CSP-Luc. Representative dot plots gated on live lymphocytes showing CD8^+^ versus CD3^+^ (left panel) and CD8^+^ versus H-2Kd CSP tetramer (left panel) expression (B) Absolute number of CD8^+^ T cells in mock-immunized mice and in mice immunized with *Pyfabb/f* ^−^ sporozoites. (C) Percentage of CSP-specific CD8^+^ T cells in mock-immunized mice and in mice immunized with *Pyfabb/f* ^−^ sporozoites. (D) Characterization of IFN-γ producing liver CD8^+^ T cells in mice immunized with *Pyfabb/f* ^−^ sporozoites (bottom panel) or mock-immunized (top panel) as determined by intracellular cytokine staining. Representative dot plots gated on live CD3^+^ lymphocytes showing CD8^+^ versus IFN-γ expression. (E) Representative dot plot gated on live CD3^+^ CD8^+^ T cells showing H-2Kd-CSP versus IFN-γ expression. (F) Absolute number of IFN-γ^+^ CD8^+^ T cells in in mock-immunized mice and in immunized with *Pyfabb/f* ^−^ sporozoites. (G) Percentage of CSP-specific CD8^+^ T cells producing IFN-γ in mock-immunized mice and in mice immunized with *Pyfabb/f* ^−^ sporozoites. (B–C and F–G) The data represents 3–5 individually analyzed mice in each group, and correspond to mean ± SD; significant differences between the mean of the mock vs. *Pyfabb/f* ^−^-immunized mice calculated using the Mann-Whitney test are indicated (* = *p*<0.05).

Next, we addressed the specificity and mechanism by which hepatocytes presenting specific epitopes are killed by malaria-specific CD8^+^ T cells after whole parasite vaccination. We isolated infiltrating liver lymphocytes from mice immunized with *Pyfabb/f* ^−^ as well as from mock-immunized mice 7 days after HTVI challenge with plasmid encoding for *Py*CSP-Luc fusion protein. The number of total and CSP-specific CD8^+^ T cells was analyzed by flow cytometry using the gating strategy shown in Figure 3A (left panel). We observed a significant increase in the number of CD8^+^ T cells present in *Pyfabb/f* ^−^-immunized mice challenged with *Py*CSP-Luc plasmid DNA vs. mock-immunized mice (Figure 3B). To determine whether these cells were specific for *Py*CSP, we used a CSP H-2Kd tetramer, as previously described [15] (Figure 3A, right panel), and established that CSP-specific CD8^+^ T cells were significantly increased in *Pyfabb/f* ^−^-immunized mice challenged with *Py*CSP-Luc plasmid DNA as compared to mock-immunized mice (Figure 3C).

IFN-γ is a critical component in CD8^+^ T cell-mediated protection induced by attenuated whole parasites [39]–[41]. We used intracellular flow cytometry analysis to determine what proportion of CD8^+^ T cells produced IFN-γ in response to GAP immunization. We saw that IFN-γ-producing CD8^+^ T cells were significantly increased in *Pyfabb/f* ^−^-immunized mice as compared to mock-immunized mice after challenge with plasmid DNA encoding for *Py*CSP-Luc (Figure 3D and F). Finally, we analyzed whether these cells were specific for CSP by using the H-2Kd tetramer described above. As shown in Figure 3E and G, the number of *Py*CSP^+^ IFN-γ^+^ CD8^+^ T cells was also significantly increased in *Pyfabb/f* ^−^-immunized mice challenged with plasmid DNA encoding for *Py*CSP-Luc as compared to mock-immunized mice. Taken together, our results suggest that a CSP-specific recall response involving IFN-γ secretion is generated during challenge of *Pyfabb/f* ^−^-immunized mice with *Py*CSP-Luc plasmid DNA, and that this response correlates with the elimination of hepatocytes that present CSP epitopes, as measured by the reduction of bioluminescence.

### Identification of *Py*Tmp21 as a potential protective antigen that reduces liver stage parasite burden

**Figure 4:**
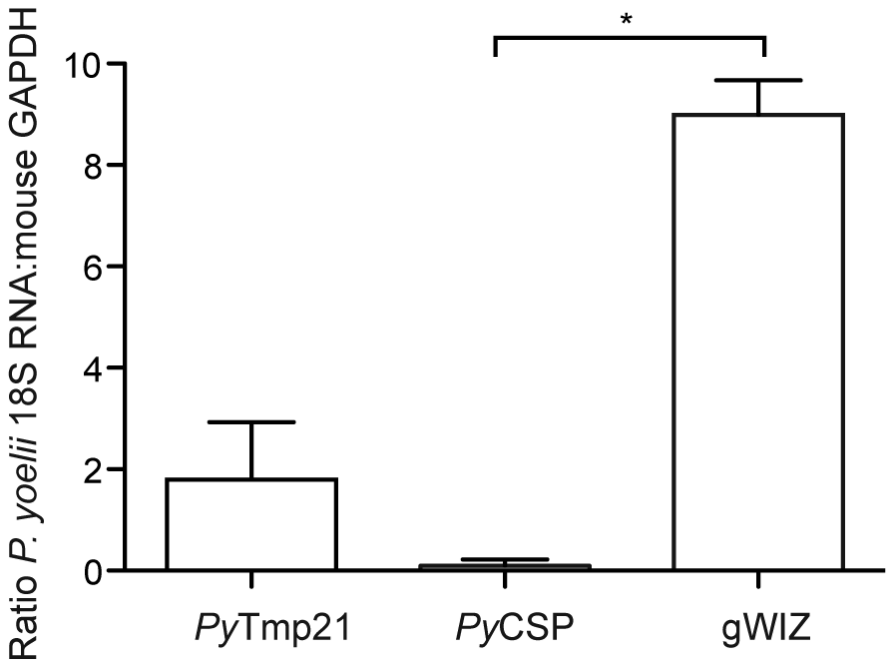
DNA vaccination of mice with *Py*Tmp21 reduces liver stage parasite burden. Shown is the average ratio of *P. yoelii* 18S RNA to GAPDH mouse housekeeping gene mRNA for 3–5 individually analyzed mice immunized with gWIZ, *Py*CSP or *Py*Tmp21 plasmid DNA. Statistical significance between the *Py*CSP or *Py*Tmp21 as compared to gWIZ plasmid was calculated using the Mann-Whitney test (* = *p*<0.05).

We previously identified PVA022 (PVX_082595) as a potential PE antigen recognized by PBMCs obtained from Duffy receptor negative donors who display naturally acquired immunity to *P. vivax* [42], in agreement with the observation that its *P. falciparum* ortholog (PF3D7_1333300) is expressed by sporozoites. To determine if this antigen can induce a protective response *in vivo*, we generated a DNA vaccine by cloning the *P. yoellii* ortholog of PVA022 (*Py*Tmp21, or PY06414) into the gWIZ vector. Empty gWIZ plasmid and gWIZ-*Py*CSP (encoding for full-length *P. yoelii* CSP) were used as negative and positive controls, respectively. BABL/c mice (5 per group) were vaccinated three times at 3-week intervals by intramuscular (i.m.) administration of 20 µg of each of these plasmids and challenged with 20,000 *P. yoelii* 17XNL sporozoites ten days after the last immunization. The inhibition of liver stage parasite development was measured by qRT-PCR 24 h later. The parasite burden was calculated as the ratio of *P. yoelii* 18S RNA to that of mouse housekeeping gene GAPDH. Vaccination with *Py*CSP reduced liver stage parasite load by 98.4%, as compared to the negative control (Figure 4). Similarly, vaccination with *Py*Tmp21 reduced parasite load by 71.8%. This result shows that immunization with a *Py*Tmp21 DNA vaccine triggers an immune response that is sufficient to reduce liver stage burden.

### *Py*Tmp21 contributes to the protective immune response elicited by *Pyfabb/f* ^−^ immunization

We next determined whether the HTVI/IVIS technique optimized with *Py*CSP can be used to validate other PE antigens as contributing to protection elicited by whole parasite vaccines by evaluating the ability of *P*yTmp21 to induce hepatocyte killing *in vivo*. To do this, we cloned the central region of *Py*Tmp21 (excluding the amino-terminal signal peptide and the carboxy-terminal trans-membrane domain) into the pHCMV-Luc vector as an amino-terminal Myc tagged, carboxy-terminal luciferase fusion protein (Figure S1A, right panel). We then tested the *in vitro* expression and luciferase signal of the fusion protein, as described above for *Py*CSP-Luc (Figure S1B–C). Although the *in vitro* expression level of *Py*Tmp21-Luc protein was much lower than that of *Py*CSP-Luc as measured by Western blot analysis (Figure S1B), the luciferase signal of the cell lysate was only one order of magnitude lower than that of *Py*CSP-Luc (Figure S1C). This could be due to differences in the stability of the fusion protein, as suggested by the strong band corresponding to luciferase in the *Py*Tmp21-luc lane of the Western blot (Figure S1B).

**Figure 5:**
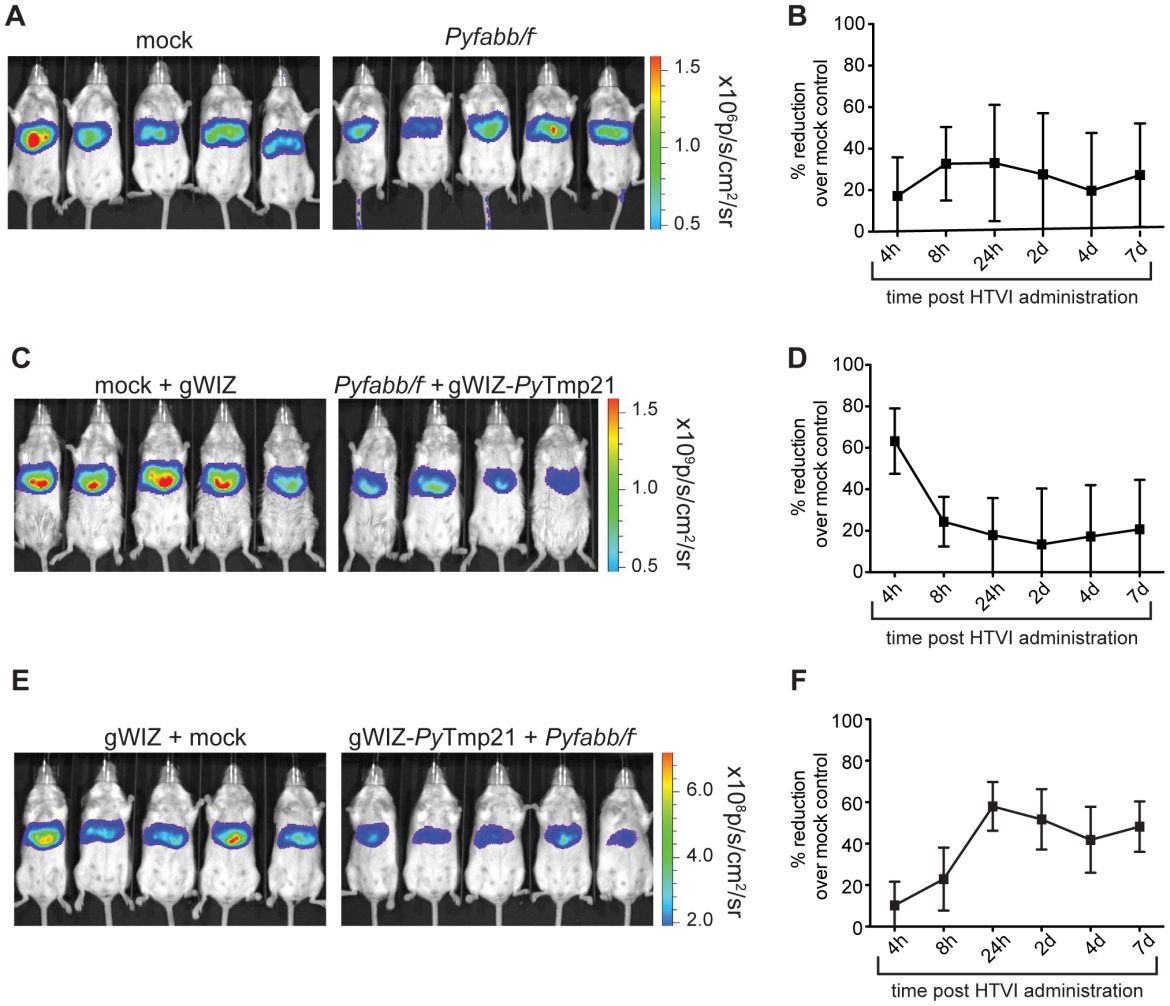
*Py*Tmp21 contributes to the protective immune response elicited by GAP vaccines. (A) Quantification of luciferase signal (shown as total flux p per second) in mice immunized twice with 50,000 *Pyfabb/f^−^* salivary gland sporozoites (right panel) or mock-immunized with salivary gland debris (left panel), challenged by HTVI with 25 µg of phCMV-*Py*Tmp21-Luc 30 days after the last immunization. Each group contained 5 mice. (B) Inhibition of luciferase signal over the course of the experiment calculated as percentage reduction vs. the mock-immunization control. The data represents 5 individually analyzed mice in each group and correspond to mean ± SD. (C) Quantification of luciferase signal (shown as total flux p per second) in mice primed with 50,000 *Pyfabb/f^−^* salivary gland sporozoites and boosted with gWIZ-*Py*Tmp21 (right panel), or mock-immunized and injected with gWIZ (left panel), challenged as described in part A. Each group contained 4 to 5 mice. (D) Inhibition of luciferase signal over the course of the experiment calculated as percentage reduction vs. the mock-immunization control. The data represents 5 individually analyzed mice in each group and correspond to mean ± SD. (E) Quantification of luciferase signal (shown as total flux p per second) in mice primed with gWIZ-*Py*Tmp21 and boosted with 50,000 *Pyfabb/f^−^* salivary gland sporozoites (right panel), or injected with gWIZ and mock-immunized (left panel), challenged as described in part A. Each group contained 5 mice. (F) Inhibition of luciferase signal over the course of the experiment calculated as percentage reduction vs. the mock-immunization control. The data represents 5 individually analyzed mice in each group and correspond to mean ± SD.

We then immunized mice with two doses of 50,000 *Pyfabb/f^−^* sporozoites each, followed by HTVI challenge with 25 µg of plasmid DNA encoding for *Py*Tmp21-Luc thirty days after the last dose. As a control, we mock-immunized mice with mosquito salivary gland debris. In contrast to the result previously obtained for *Py*CSP using this strategy, in this case we failed to observe a reduction in the luciferase signal upon challenge (Figure 5A and B). Therefore, in spite of our preliminary data showing that immunization with *Py*Tmp21 as a DNA vaccine can protect mice against malaria infection, HTVI challenge with *Py*Tmp21 after whole parasite vaccination did not result in hepatocyte destruction. This observation could be explained by a recent report suggesting that exposure to whole parasite vaccines results in a bias of the immune response towards the immunodominant CSP, and away from less abundant antigens expressed during PE stages [43].

To overcome this obstacle, we designed a heterologous prime/boost strategy consisting of an initial vaccination with a single dose of 50,000 *Pyfabb/f^−^* sporozoites, followed by boosting with plasmid DNA encoding for *Py*Tmp21 (gWIZ-*Py*Tmp21). Using this method, we observed a 60% reduction in luciferase signal 4 h after HTVI challenge of *Pyfabb/f^−^*-primed, gWIZ-*Py*Tmp21 boosted mice with plasmid DNA encoding for *Py*Tmp21-Luc, as compared to mock-primed, gWIZ-boosted mice (Figure 5C and D). This initial high level of inhibition of luciferase signal diminished to ∼20% at the 8 h time point, and remained at this level until 7 d post challenge (Figure 5D). This result suggests that priming with *Pyfabb/f^−^* elicits *Py*Tmp21-specific T cells that can be boosted by the subsequent administration of plasmid DNA encoding for this antigen, resulting in the killing of hepatocytes that display the antigen upon HTVI challenge.

Finally, we also tested the effect of priming with plasmid DNA encoding for *Py*Tmp21, followed by boosting with a single dose of *Pyfabb/f^−^* parasites. Interestingly, in contrast to the fast response observed when priming with attenuated whole parasites followed by boosting with plasmid DNA, in this case we observed a delayed reduction in the luciferase signal (Figure 5E and F). In other words, we saw an initial 10% reduction 4 h after HTVI challenge of gWIZ-*Py*Tmp21-primed, *Pyfabb/f^−^-*boosted mice, as compared to gWIZ-primed, mock-boosted mice, which then steadily increased to 60% at 24 h and remained constant at approximately 50% until 7 d post challenge (Figure 5F). Taken together, our data suggest that although immunization with whole parasites was not sufficient to generate a significant T-cell based response to *Py*Tmp21, likely because of the bias of the immune response towards immunodominant CSP, the use of heterologous immunization strategies that combine whole parasite and DNA vaccines generates a detectable immune response against *Py*Tmp21, validating it as a PE antigen that contributes to the protective effect of whole parasite vaccines.

## Discussion

Ideal anti-infection malaria vaccines should target the silent pre-erythrocytic stages, blocking sporozoite invasion and/or subsequent development in the hepatocyte, hence preventing malaria infection and disease [2]. In fact, the most effective malaria vaccines tested to date are all based on attenuated parasites [8], [10], [44]. The use of immunization strategies that result in late LS or early BS arrest results in the development of immunity against a broad spectrum of antigens expressed by LS-infected hepatocytes and as consequence, in enhanced protection against sporozoite challenge [19], [44].

Human and mouse studies have shown that immunization with whole parasites elicits both humoral and T-cell responses, and that the bulk of this response is directed towards the immunodominant sporozoite surface protein CSP [27], [43], [45]. Thus, subsequent boosts with whole parasites will repeatedly enhance the response to CSP, leading to significant reductions of hepatocyte invasion by spz and LS parasite load in the liver. As an unintended consequence, however, this immunization strategy prevents the development of robust T cell responses against non-CSP antigens expressed by LS parasites [43], perhaps explaining why extremely high doses of irr-spz are required in order to reach the peak of anti-CSP immunity that allows efficient inhibition of hepatocyte invasion by spz after challenge with wt *P. falciparum* spz [10]. Although CSP-specific CD8^+^ T cell responses were induced by *P. yoelii* GAP in this study, CSP has been proven to be dispensable in C57BL/6 mice following vaccination with *P. berghei* sporozoites attenuated through irradiation [23], and was only marginally involved in protection of BALB/c mice immunized with *P. berghei* under chloroquine coverage [25]. These results highlight the importance of identifying pre-erythrocytic antigens other than CSP that contribute to the protection elicited by attenuated whole parasite vaccines, which will be important in designing effective immunization strategies that result in a broad immune response that can control LS parasite infections that might have escaped CSP-dependent responses.

To enable the identification of *bona fide* LS antigens that contribute to the protection induced by whole parasite immunization, we took advantage of the *P. yoelii* FabB/F gene knock-out GAP (*Pyfabb/f^−^*) that arrests late in the LS parasite development [46]. Immunization with *Pyfabb/f^−^* induces sterile long-term protection and a broad CD8^+^ T cell response [19], therefore allowing the induction of immunity against LS antigens. Thus, this parasite is ideal model for identifying LS antigens that contribute to *Pyfabb/f^−^* -induced CD8^+^ responses. Moreover, to measure the ability of antigen-specific CD8^+^ T cells to eliminate hepatocytes *in vivo* after immunization of mice with *Pyfabb/f^−^*, we utilized a highly efficient, non-viral method for delivery of DNA into the liver, known as HTVI. This technique consists of the rapid injection of a large volume of plasmid DNA into the tail vein, resulting in uptake of the DNA into the cytoplasm of liver cells [35], and has been widely used in recent years to deliver DNA and RNA for gene function, gene therapy and for establishment of disease animal models (reviewed in [47]). Although HTVI is a valid approach to deliver antigens directly into the liver, it has been recently shown that it does not constitute and effective vaccination strategy because it leads to defective CD8^+^ responses [48]. In contrast, this method has been used to show that immunization of mice with adenovirus vectors that encode for the well-established *P. yoelii* PE antigens *Py*CSP or *Py*CelTOS, followed by HTVI challenge with the same proteins as luciferase fusions results in significant reductions in the luciferase signal in the liver [49].

In this study, we first used CSP as a model protein to optimize the HTVI challenge of *Pyfabb/f^−^-*immunized mice. We observed that HTVI successfully delivered plasmid DNA into hepatocytes, resulting in the expression of luciferase-tagged *Py*CSP (Figure 1 and Figure S2). Importantly, mice immunized with two doses of *Pyfabb/f^−^* sporozoites and challenged with plasmid DNA encoding for luciferase-tagged *Py*CSP displayed a reduced luciferase signal (Figure 1), suggesting that immunization with whole parasites can induce a CSP-specific immune response that is able to eliminate hepatocytes that present CSP on their surface. Furthermore, depletion of CD8^+^, but not of CD4^+^ T cells, abrogated the suppression of the luciferase signal (Figure 2), demonstrating that hepatocyte killing is CD8^+^ dependent. We also showed an increase in the level of total and IFN-**γ** producing CSP-specific CD8^+^ T cells in the liver of mice immunized with *Pyfabb/f^−^* and challenged with *Py*CSP-Luc (Figure 3). These results agree with previous data from our group and others suggesting that the elimination of malaria-infected hepatocytes is mediated by CD8^+^ T cells [14], [16], [28].

Next, as a proof of concept of the ability of this method to identify novel LS antigens that associate with the protective immunity induced by whole parasite vaccination, we used it to test *Py*Tmp21. The *P. vivax* orthologue of *Py*Tmp21 was originally identified as a potential novel PE antigen recognized by PBMCs of Duffy receptor negative donors with naturally acquired malaria immunity to *P. vivax* [42], who do not support blood stage *P. vivax* infection as a result of the mutation. We showed that *Py*Tmp21 elicits functional immunity that significantly reduces LS parasite burden after challenge with wt *P. yoelii* spz (Figure 4). However, we were initially unable to confirm *Py*Tmp21 as an antigen that contributes to the protection elicited by *Pyfabb/f^−^* sporozoites using the immunization strategy that worked for *Py*CSP (Figure 5A and B). This result agrees with recent data suggesting that repeated exposure to whole parasite vaccines results in a bias of the immune response towards the immunodominant CSP, and away from less abundant antigens expressed during PE stages [43].

To overcome this obstacle, we explored different heterologous priming/boost immunization strategies. First, we vaccinated mice with a single dose of *Pyfabb/f^−^* sporozoites followed by boosting with plasmid DNA encoding for *Py*Tmp21. In contrast to the initial negative result obtained for *Py*Tmp21 upon repeated immunization with *Pyfabb/f^−^*, this strategy resulted in reduction of the luciferase signal upon HTVI challenge with *Py*Tmp21 as a luciferase fusion (Figure 5C and D). Vaccination of mice with plasmid DNA encoding for *Py*Tmp21 followed by boosting with a single dose of *Pyfabb/f^−^* also resulted in a reduction of luciferase signal upon challenge with *Py*Tmp21-Luc plasmid DNA (Figure 5E and F). These data suggest that *Py*Tmp21 is expressed by *Pyfabb/f^−^* parasites, and that it contributes to protection induced by whole parasite vaccination. Interestingly, the kinetics of these two immunization strategies were strikingly different: whereas priming with *Pyfabb/f^−^* followed by boosting with plasmid DNA resulted in 60% reduction of luciferase signal 4 h post challenge, which then declined to about 20% for the duration of the experimental time course, priming with DNA followed by boosting with *Pyfabb/f^−^* yielded an initially low response that only peaked at 60% 24 h post challenge and remained fairly constant thereafter (Figure 5D and F). It is possible that these different responses could be due to the different location of memory T cells elicited by DNA and whole parasite vaccinations [50]. Priming the immune system with a DNA vaccine administered through the i.m. route results in a T cell response that is localized to peripheral sites, so that upon boosting with *Pyfabb/f^−^* these cells have to migrate to the liver in order to respond to the HTVI challenge, resulting in a delayed response. In contrast, priming with *Pyfabb/f^−^* results in a liver infection that induces tissue resident T cells, so that boosting with DNA leads to a rapid expansion of these cells and a quick induction of cytotoxic effector cells that can destroy hepatocytes presenting specific antigens.

Based on our observation that elimination of *Py*CSP-Luc signal is abrogated in the absence of CD8^+^ cells, in addition to previous data from our group and others that demonstrate the essential role of CD8^+^ T cells in controlling pre-erythrocytic malaria infection [14], [16], [17], [28], we hypothesize that *Py*Tmp21-expressing hepatocytes are most likely eliminated by mechanisms that depend on CD8^+^ T cells. Future studies will be aimed at determining the specific mechanism involved in the reduction of luciferase signal observed upon challenge with *Py*Tmp21-Luc of mice immunized with heterologous strategies that combine whole parasites and DNA vaccines.

In conclusion, using CSP as a model, we were able to confirm that the HTVI/IVIS method enables the detection of hepatocytes that are killed as a consequence of presenting specific parasite antigens, and that this killing depends on CD8^+^ T cells. Furthermore, the data presented herein show that the use of a heterologous immunization strategy coupled with the HTVI/IVIS method constitutes a powerful tool to validate pre-erythrocytic antigens that contribute to the protection elicited by whole parasite vaccines. In particular, we confirmed that *Py*Tmp21, which we previously identified as a novel pre-erythrocytic antigen, contributes to the protective immunity elicited by whole parasite vaccinations. Ultimately, the method described herein can be used to validate new malaria vaccine candidates and increase our understanding of how whole parasite immunization protects against malaria, thus paving the way for intelligent vaccine design.

## Supporting Information

Figure S1 Cloning and expression of *Py*CSP and *Py*Tmp21 luciferase-fusion proteins. (A) Representation of the structure of *Py*CSP-Luc and *Py*Tmp21-Luc. The diagram shows the amino-terminal Myc tag, the carboxy-terminal luciferase fusion protein, and the regions and elements of the *P. yoelii* proteins included in the constructs. (B–C) Assessment of expression of the fusion proteins *ex vivo*. (B) Cell lysates purified from COS-7 cells transfected with phCMV-*Py*CSP-Luc, phCMV-*Py*Tmp21-Luc, phCMV-Luc or not transfected were separated by protein gel electrophoresis and transferred to a nitrocellulose membrane. Luciferase fusion proteins were identified by probing the membrane with a polyclonal anti-luciferase antibody by Western blot. (C) Cell lysates prepared as described in part B were assayed for luciferase activity 48 hours after transfection, using a luminometer.
(TIF)

Figure S2 Expression of *Py*CSP by hepatocytes and liver lymphocytes. Representative dot plots showing the percentage of *Py*CSP positive cells gated on hepatocytes (A), Kupffer cells (B); dendritic cells (C), Sinusodial Liver Endothelial Cells (D), T cells (E) and B cells (F), obtained from mice injected with phCMV-Luc (left panel, n = 2) or phCMV-*Py*CSP-Luc (middle panel, n = 3). The graphs on the right show the data for all mice. The horizontal bar indicates the mean.
(TIF)
