# Supplementary figures and images for: Identification of Pre-Erythrocytic Malaria Antigens That Target Hepatocytes for Killing In Vivo and Contribute to Protection Elicited by Whole-Parasite Vaccination
Source: PLoS One. 2014 Jul 15;9(7):e102225. doi: 10.1371/journal.pone.0102225 (PMC4099202; doi:10.1371/journal.pone.0102225)

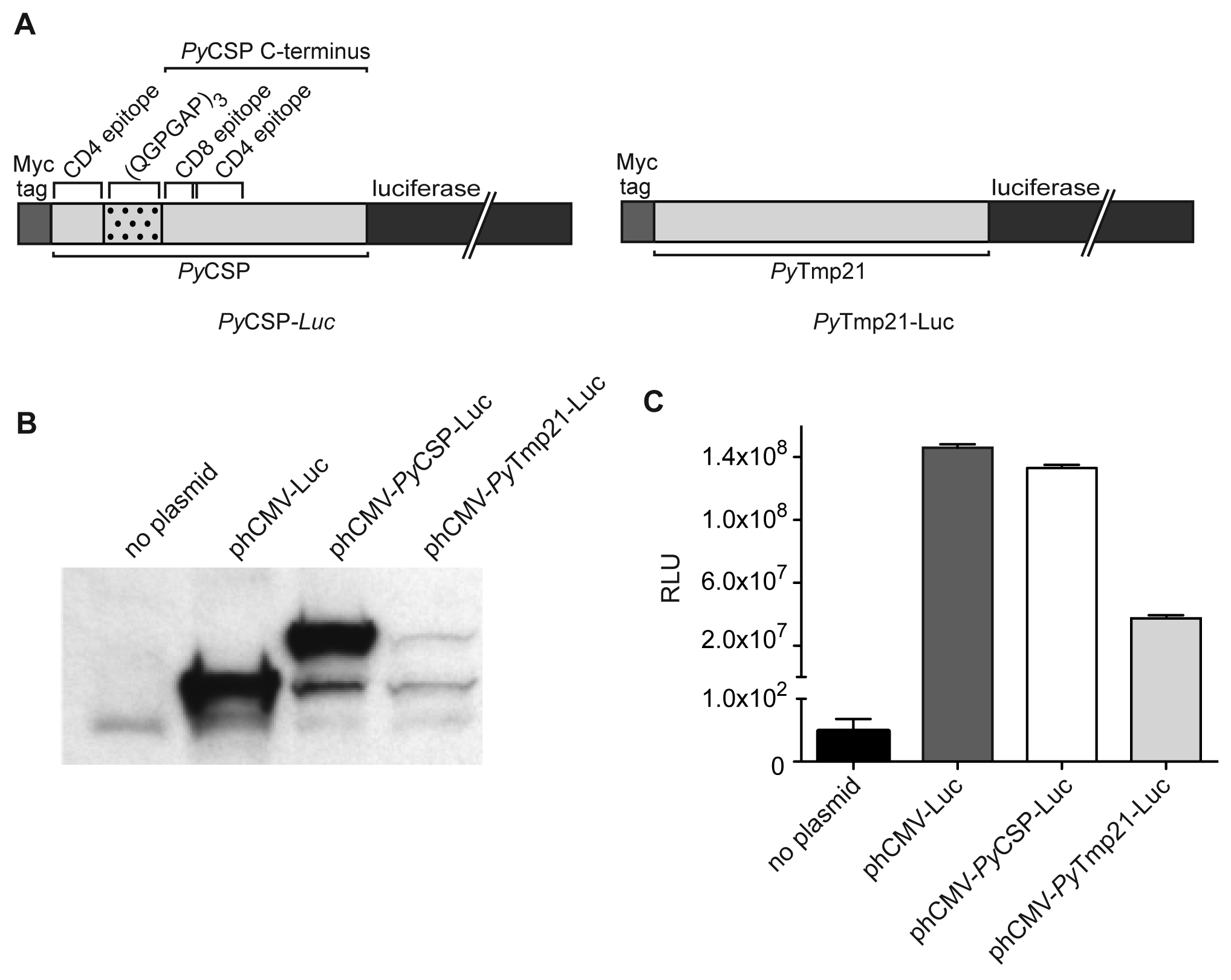

Supplement: Figure S1 — Cloning and expression of PyCSP and PyTmp21 luciferase-fusion proteins. (A) Representation of the structure of PyCSP-Luc and PyTmp21-Luc. The diagram shows the amino-terminal Myc tag, the carboxy-terminal luciferase fusion protein, and the regions and elements of the P. yoelii proteins included in the constructs. (B–C) Assessment of expression of the fusion proteins ex vivo. (B) Cell lysates purified from COS-7 cells transfected with phCMV-PyCSP-Luc, phCMV-PyTmp21-Luc, phCMV-Luc or not transfected were separated by protein gel electrophoresis and transferred to a nitrocellulose membrane. Luciferase fusion proteins were identified by probing the membrane with a polyclonal anti-luciferase antibody by Western blot. (C) Cell lysates prepared as described in part B were assayed for luciferase activity 48 hours after transfection, using a luminometer. (TIF) [file pone.0102225.s001.tif]

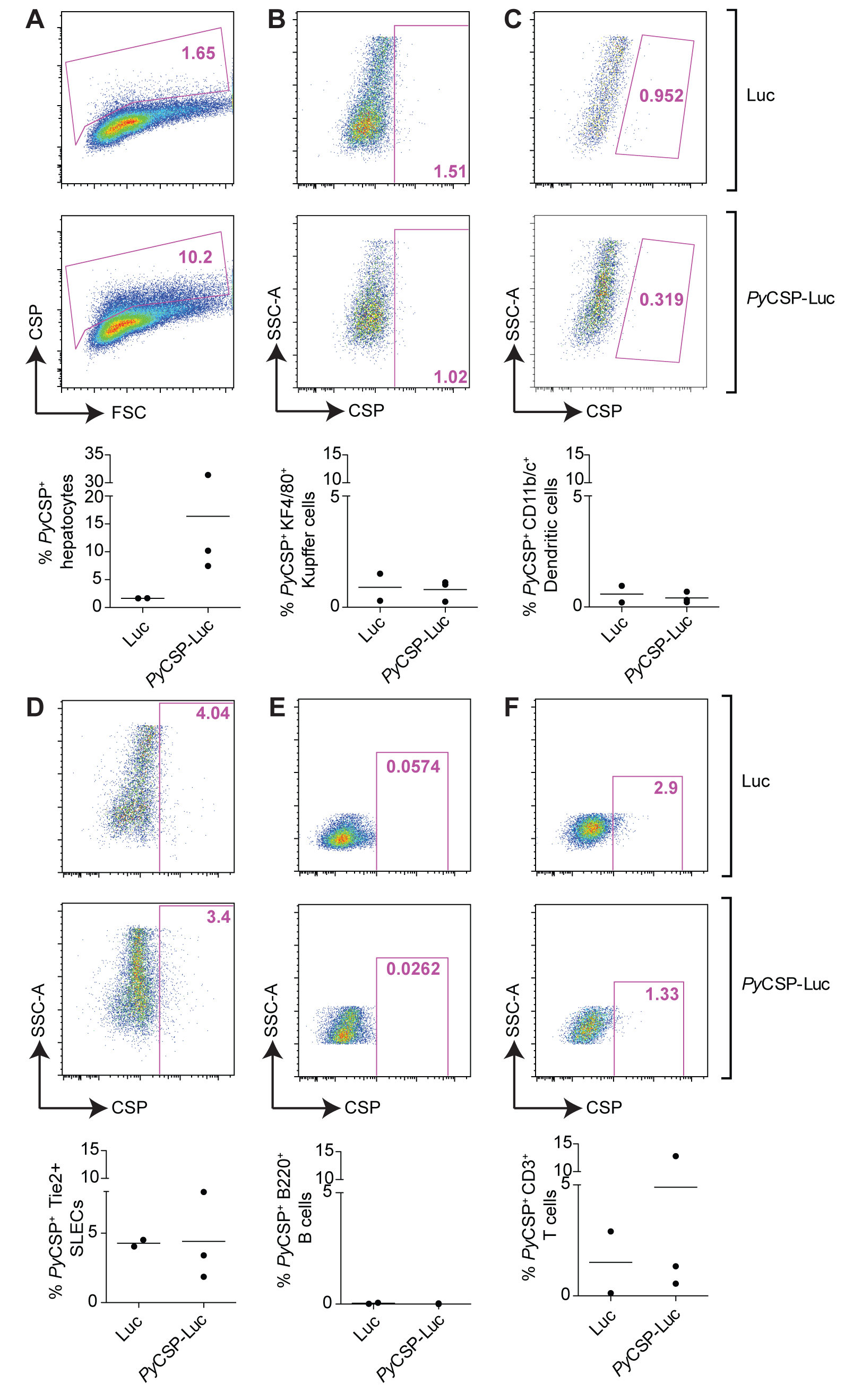

Supplement: Figure S2 — Expression of PyCSP by hepatocytes and liver lymphocytes. Representative dot plots showing the percentage of PyCSP positive cells gated on hepatocytes (A), Kupffer cells (B); dendritic cells (C), Sinusodial Liver Endothelial Cells (D), T cells (E) and B cells (F), obtained from mice injected with phCMV-Luc (left panel, n = 2) or phCMV-PyCSP-Luc (middle panel, n = 3). The graphs on the right show the data for all mice. The horizontal bar indicates the mean. (TIF) [file pone.0102225.s002.tif]
